# Ductal Carcinoma in Situ: A Detailed Review of Current Practices

**DOI:** 10.7759/cureus.37932

**Published:** 2023-04-21

**Authors:** Dheer S Kalwaniya, Madhur Gairola, Sumedha Gupta, G Pawan

**Affiliations:** 1 General Surgery, Vardhman Mahavir Medical College and Safdarjung Hospital, New Delhi, IND; 2 Obstetrics and Gynaecology, Vardhman Mahavir Medical College and Safdarjung Hospital, New Delhi, IND

**Keywords:** dcis management, risk factors for breast cancer, ductal carcinoma in-situ, dcis, dcis prevalence

## Abstract

Ductal carcinoma in situ is a challenge for breast surgeons, beginning with its difficult radiological detection and continuing with its contentious multimodal treatment and management. It is becoming more common as a result of widespread screening mammography and usually manifests as a cluster of calcifications. Patients are usually asymptomatic or present with a small, palpable lump. It is, however, a premalignant lesion that has the potential to progress to invasive carcinoma and is treated similarly with multimodal therapy. Treatment options currently include total or simple mastectomy with sentinel lymph node biopsy or lumpectomy with radiation. Tamoxifen and human epidermal growth factor receptor two suppression therapy are examples of adjuvant therapy. A review of consensus guidelines and literature was performed, in which we included the available online literature on the concerned topic from 2000-2022. This article is not a complete review of all the available literature; rather, it is a comprehensive review of the topic and its current management guidelines.

## Introduction and background

Ductal carcinoma in situ (DCIS) is a premalignant neoplasia of mammary ducts that is confined to the lumen of the ducts, is lined by a layer of semicontinuous myoepithelial cells, and does not breach the basement membrane. DCIS was uncommon before 1980, but it now accounts for more than 20% of all new breast cancer diagnoses in the US, with over 60,000 new cases diagnosed each year [1]. In the past, patients would present with a large palpable mass that tended to carry a higher risk of conversion into invasive breast carcinoma, and thus DCIS was labeled as a potential premalignant lesion. Currently, with the advent of newer diagnostic modalities and screening mammography, DCIS is usually detected in its earlier stage, commonly in the non-comedo form. One of the most significant risk factors for DCIS is advanced age. DCIS rates rise with age, from 0.6 per 1,000 screening examinations in women aged 40-49 to 1.3 per 1,000 screening examinations in women aged 70-84 [1,2]. DCIS shows microcalcifications on mammography, with detection by palpation accounting for less than 10% of cases [3]. With the advent of molecular biology and its correlation with the development of neoplasms, the progression of DCIS is better understood. Various studies have been conducted with the aim of characterizing DCIS and invasive breast carcinoma at the molecular level, and it has been found that the two have genetic similarity and likely a common origin [4-8]. Although the proportion of DCIS that progresses to invasive breast carcinoma varies greatly, studies show that after a long period of observation, 20%-30% of DCIS may progress without definitive treatment [8,9]. In a study done by Steven A. Narod et al. in 2015, it was observed that DCIS has a 3.3% mortality rate specific to breast cancer [10]. However, whether the deaths that occurred were caused by the progression of DCIS into recurrent invasive breast carcinoma or by the DCIS itself having indolent malignant potential remains unclear [10]. As a result, detecting and treating DCIS is routinely advised to reduce long-term breast cancer-specific mortality. There are enormous variations in DCIS treatment patterns [11]. Treatment options currently recommended by the National Comprehensive Cancer Network (NCCN) include mastectomy with sentinel lymph node biopsy, lumpectomy with radiation, or lumpectomy alone with the potential addition of Tamoxifen and aromatase inhibitors in post-menopausal women with hormone receptor-positive DCIS [8,9].

Randomized trials show that lumpectomy combined with radiation therapy (RT) reduces DCIS locoregional recurrence but is insufficiently powered to detect a difference in breast cancer-specific or overall survival [8,9]. The overall aim of the addition of radiation as adjuvant therapy was to prevent recurrence because it was observed that most of the mortality was due to recurrent disease that used to evolve as an invasive breast carcinoma. Four clinical trials were carried out to determine the role of radiotherapy after the lumpectomy in the treatment of DCIS. In the meta-analysis of these trials, it was observed that there was a halving in the risk of local recurrences (both in situ and invasive) after radiotherapy [12].

Adjuvant radiation therapy post breast conserving surgery was found effective in lowering the risk of local recurrence [12]. Rationale was sought behind treating every DCIS with mastectomy if it was indolent and did not cause any metastasis. To seek a tailored approach to therapy, the role of risk stratification came into play to differentiate the histologic variants of DCIS and subject them to association with the course and aggressiveness of the disease. The advent of histologic classification of DCIS can be traced back to the pre-mammographic era, when it was introduced by Allred DC in his study, where he divided DCIS into two subtypes: the comedo group, which is the large-cell, more aggressive form, and the non-comedo group, which is the small-cell, less aggressive form [13]. Previously, most of the encountered cases of DCIS were large, irregular tumorous masses arising from rapidly dividing cells within the ducts with the development of necrosis. They usually formed a large palpable mass and were referred to as comedo because, when the tumor specimen post-excision was squeezed, necrotic material oozed out, resembling comedone in acne. The other rarely encountered variants of DCIS before mammography were not palpable or grossly visible and were classified primarily based on their predominant microscopic pattern, which included papillary, cribriform, solid, or micropapillary, and were collectively referred to as non-comedo [13]. This classification system tells us about the likely extent of disease; for example, the micropapillary variant of DCIS is more likely to be multi-quadrant. In some series, comedo-type DCIS is usually found on mammography, whereas cribriform disease is identified clinically [2]. The reproducibility of this system of categorization based solely on growth patterns is, however, problematic. 

Lesions most frequently show a mixture of architectures, which is seen nearly twice as frequently as the second most common solid pattern [14]. Furthermore, even a single duct space may exhibit an architectural pattern that is difficult to classify. Newer systems are typically based on nuclear grade, which is less commonly mixed (15.7%), with some also incorporating the presence or absence of luminal necrosis [15,16]. The currently used and widely accepted histologic classification is the Van Nyhus classification. The Van Nyhus prognostication index (VNPI) categorizes DCIS patients to guide treatment decisions. Based on the patient's age, tumor size, tumor growth patterns (histological grade), and the amount of healthy tissue surrounding the tumor after removal, the index predicts the risk of cancer recurrence [17]. Patients are classified into three groups based on the sum of their scores from each of these factors: breast-conserving surgery (BCS) without radiotherapy is recommended for low-risk patients (total VNPI score of 4-6), BCS with radiotherapy is recommended for intermediate-risk patients (total VNPI score of 7-9), and mastectomy is recommended for high-risk patients (total VNPI score of 10-12). [17]. There are still a lot of discrepancies as regards the treatment, and various studies have been conducted. The controversy over DCIS being a potential premalignant condition or not remains, with no current universal consensus on the histological grading of the disease. The present review paper thus emphasizes the epidemiology, pathology, diagnosis, grading, treatment modalities, and follow-up of DCIS.

## Review

Epidemiology

Since the introduction of mammographic screening in the early 1980s, the incidence of DCIS has increased dramatically over the past three decades. Each year, more than 50,000 women in the United States are diagnosed with DCIS; among newly diagnosed breast tumors, this accounts for around 18%-25% [18,19]. However, as per the Surveillance, Epidemiology, and End Results (SEER) registry, the incidence of DCIS from 2000-2014 remained stable. In a study conducted by Marc D. Ryser, the author used SEER registry data and computed age-specific, race-specific, and mammogram relations with the incidence of DCIS from 2000-2014 and found that DCIS incidence increased by 1.3% (P = 0.001) and 0.6% (P = 0.02) per year in the age groups 20-44 years and 45-55 years, respectively [20]. Although it remained stable among white women, DCIS incidence increased by 1.6% (P 0.001) and 1.0% (P = 0.002) per year among black women and women of other races, respectively [20]. Apart from women aged 40-49 years and black women, who experienced an increase in DCIS incidence despite stagnant and decreasing mammography uptake, respectively, mammography uptake correlated well with DCIS incidence. In a study of women aged 50-69 years from 15 screening programs across 12 International Cancer Screening Network countries between 2004 and 2008, the overall incidence of DCIS averaged 16% (0.82 per 1,000 examinations), with incidence being highest in the United States (24%; 95% CI: 22-25%) and lowest in Finland (9%; 95% CI: 8-10%) [21]. Studies were conducted to look for the hereditary pattern of DCIS, and it was observed that germline mutations of BRCA1 and BRCA2 carried an increased risk of having DCIS; however, a history of DCIS in a first-degree relative could not infer a major risk. In an analysis of the Million Women Study in the United Kingdom, Reeves et al. discovered no difference in the increased risk that a first-degree family history imparted for DCIS or invasive breast cancer (RR=1.56 and 1.60, respectively) [22]. Reinier et al. discovered that the risk of premenopausal versus postmenopausal DCIS was unaffected by family history (RR=1.9; 95% CI: 1.2-2.8; and RR=1.4; 95% CI: 1.0-2.0 for pre- and postmenopausal women, respectively) [23]. Claus et al. examined 369 DCIS patients’ BRCA 1 and BRCA 2 mutation status. When compared to rates found in patients with invasive breast cancer, they found that three patients (0.8%) and nine patients (2.45%) each had BRCA1 and BRCA2 mutations, respectively [24].

DCIS in Indian scenario

The rise in awareness and inclusion of various policies by the Indian healthcare system has led to an increase in the detection of cases of breast cancer now at an early stage. The data regarding the prevalence and incidence of various morphological variants of breast carcinoma is not available; however, there are hospital-based studies that have calculated the prevalence of various morphological variants of breast carcinoma. There was a hospital-based retrospective study done in 2005 by Sunita Saxena et al. in which they collected biopsy reports of 569 cases operated on from 1989-2003 and found that invasive ductal carcinoma, not otherwise specified, was the commonest (86.9%), whereas DCIS was observed in six cases (1.1%) [25]. This, however, won't represent the data among the population, and thus studies at a larger scale or the inclusion of data based on morphological variants in the national cancer registry might give a more appropriate overview. 

Progression into invasive breast carcinoma

DCIS is defined as a neoplastic proliferation of glandular epithelium confined within the lumen of ducts that have not breached the basement membrane. The definition changes to invasive ductal carcinoma as the basement membrane is breached. Initially presented as a palpable lump in the breast in older women, DCIS is currently increasingly picked up in its earlier preclinical stages in women younger than 50 years due to an increase in screening mammography. DCIS is currently referred to as a nonobligate precursor of invasive breast carcinoma. Although the natural course of DCIS is unknown, it is believed that many lesions with low-grade cytologic characteristics and small sizes may have a limited chance of developing into invasive carcinoma. Estimating the progression of untreated DCIS is difficult because current standards of care require most patients with any DCIS subtype, grade, or size to have excision to negative margins, usually with the addition of RT if undergoing lumpectomy and hormonally targeted therapies if indicated [26].

Diagnosis

**Figure 1:**
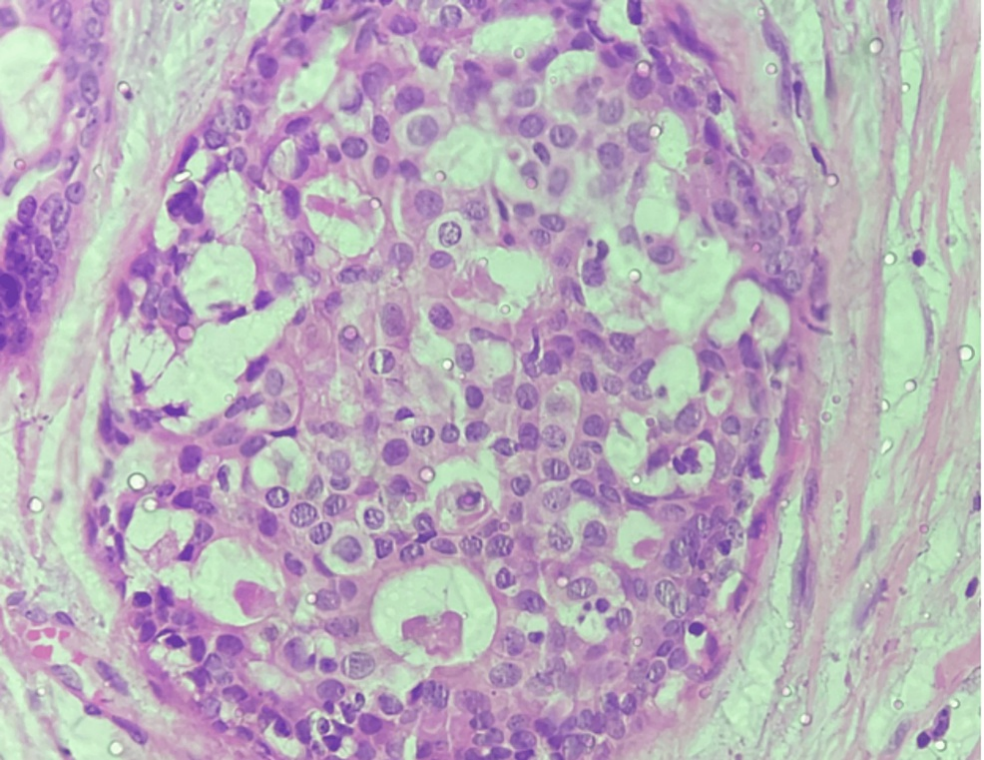
Hematoxylin and eosin stain at 40X shows cribriform-type ductal carcinoma in situ (DCIS). Histopathological examination of a post-operative specimen from a diagnosed case of DCIS. Image is obtained from the Department of Pathology, Vardhman Mahavir Medical College and Safdarjung Hospital, New Delhi.

**Figure 2:**
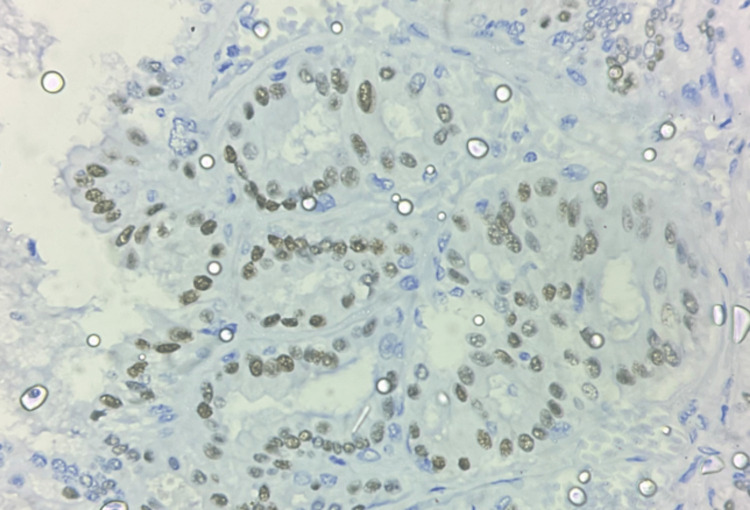
Immunohistochemistry at 40X shows ER positivity in DCIS. Histopathological examination of a post-operative specimen from a diagnosed case of DCIS. Image is obtained from the Department of Pathology, Vardhman Mahavir Medical College and Safdarjung Hospital, New Delhi.

**Figure 3:**
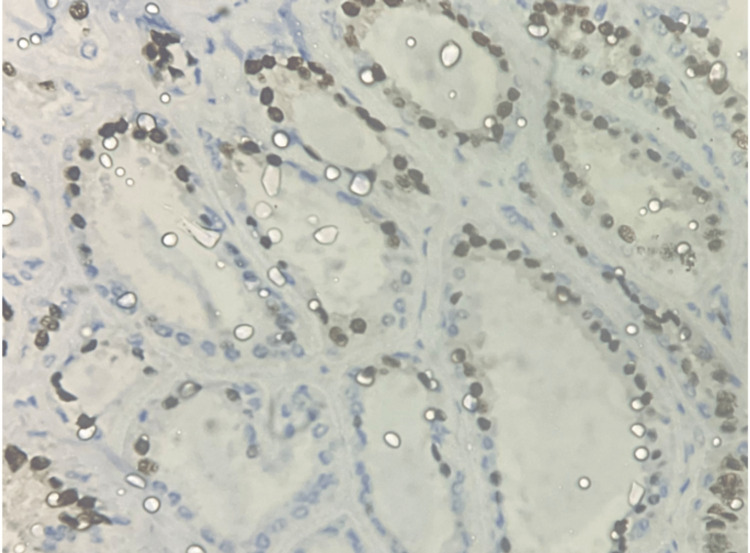
Immunohistochemistry at 40X, showing PR positivity in DCIS. Histopathological examination of a post-operative specimen from a diagnosed case of DCIS. Image is obtained from the Department of Pathology, Vardhman Mahavir Medical College and Safdarjung Hospital, New Delhi.

As mentioned earlier, there was a 500% increase in the diagnosis of DCIS after the introduction of screening mammography [27]. DCIS was diagnosed before mammographic screening as an incidental finding or when it was palpable, which is uncommon. Calcifications and, much less frequently, a mass, distortion, or focal asymmetry are features of DCIS mammographic imaging. Screening mammography is a perfect tool for preclinical diagnosis because DCIS is rarely palpable [28,29]. Mammography has a sensitivity of 87%-95% for detecting DCIS [28,29]. Although DCIS can manifest as a mass or architectural distortion, microcalcifications are the most common mammographic finding. Calcifications are present in 90% of DCIS lesions, and 80% manifest as calcifications with no other mammographic finding [30]. In a study, Stomper et al. looked at malignant mammographic calcifications without a corresponding parenchymal lesion and found that pure DCIS was present in 65% of cases, DCIS with a focus of invasion was present in 32% of cases, and invasive carcinoma alone was present in 4% of cases [31]. If suspicious calcifications are accompanied by an asymmetry or mass, they are more likely to be caused by invasive carcinoma or DCIS combined with invasive carcinoma than by DCIS on its own [31]. According to Farshid et al., when DCIS formed parenchymal lesions without radiographically visible calcifications, it was more frequently low grade, and when calcifications were present, it was more frequently high grade [32]. The parenchymal finding was caused by periductal fibrosis and chronic inflammation. The papillary component was present in most of the discrete masses [32]. The technological advancement introduced digital mammography and digital breast tomosynthesis. Studies were conducted in search of the most specific and sensitive diagnostic tool. However, no significant change in sensitivity was observed [33-35]. Later, ultrasonography was the preferred mode of imaging for screening breast cancer, but it showed decreased sensitivity for DCIS when compared to mammography. The ACRIN 6666 trial was conducted to determine the efficacy of ultrasonography as a screening modality for breast cancer, which was compared with other available imaging modalities. In this study, six women were diagnosed with DCIS, but only one case was detected by ultrasonography [36]. MRI of the breast was found to be highly sensitive for DCIS, with a sensitivity rate of 92% compared to the mammography sensitivity rate of 56% [37,38]. Non-mass enhancement is the most common morphologic appearance of DCIS on MRI, and it has been reported in 60%-80% of cases. Aside from the overall higher sensitivity, the benefits of MRI include increased sensitivity in dense breasts, better assessment of multicentricity, and better estimation of DCIS size [38]. Because MRI more accurately assesses the extent of disease than mammography, it may be useful in presurgical planning. The authors of the Comparative Effectiveness of MR Imaging in breast cancer trial, however, found no reduction in re-excision rate with preoperative MRI [39]. However, MRI has its own disadvantages, including a high cost and a high rate of false negatives. Imaging has inter-observer bias and thus requires pathological correlation to make a diagnosis. The histopathological correlation of DCIS also helps us identify the nature of the tumor and its aggressiveness. Various pathologists have tried classifying DCIS based on its histopathologic features; however, no universal consensus has been formed, and thus this area remains subject to further research. DCIS histology is distinguished by cohesive, clonal-appearing epithelial cells with prominent cell borders. DCIS, however, exhibits significant histologic heterogeneity, with a diverse range of architectural and cellular patterns such as comedo, cribriform, solid, papillary, micropapillary, clinging, apocrine, and clear-cell types. DCIS is currently graded as low, intermediate, or high nuclear grade, with documentation of necrosis, cell polarization, and prominent architectural pattern(s). Figures 1-3 show the histopathological features of DCIS with estrogen receptor (ER) and progesterone receptor (PR) IHC taken from the Department of Pathology of our institute.

Molecular biology and tumor markers

DCIS has significant variability in biomarker profiles and genetic aberrations, which is consistent with histological heterogeneity. Low nuclear grade DCIS almost always lacks human epidermal growth factor receptor two (HER-2)/neu protein overexpression or gene amplification but almost always exhibits diffuse strong ER and PR expression [40]. High-nuclear-grade DCIS, in contrast, can be ER/PR positive or negative and HER-2/neu positive in 60%-80% of cases [41]. Low-grade DCIS is defined by chromosomal losses at 16q and 17p and gains at 1q, whereas high-grade DCIS has more numerous and variable alterations, including losses at 11q, 14q, 8p, and 13q and gains at 17q, 8q, and 5p [40,41,42]. On pathological specimens of DCIS, ER, and, to a lesser extent, PR, expression is routinely assessed to predict clinical response to hormonal therapy, such as selective ER modulators. Although the role of HER-2 status in women with DCIS is unclear, the results of the ongoing NSABP B-43 trial will shed some light on its significance. In this study, the author compares ipsilateral breast cancer recurrences in women with HER-2-positive DCIS who received lumpectomy, RT, and Trastuzumab to women who received lumpectomy and RT alone [43].

Current management guidelines

With increasing detection rates of DCIS and the available modes of treatment, it is difficult for a surgeon to decide what treatment option will suit a patient best. Various clinical trials have been performed to prevent overtreatment of the disease, which is otherwise labeled as a benign proliferative disease, and increase the quality of life. After reviewing various trials and meta-analyses, NCCN has updated guidelines on management from time to time, and the most recent is stated as follows:

Workup

It is recommended to test for ER status in DCIS to determine the benefit of adjuvant endocrine therapy for risk reduction. It is not recommended to test for HER-2 status [44].

The MRI’s role remains unclear. The NCCN Panel advises using breast MRI for DCIS only when additional information is required during the initial workup, noting that MRI has not been shown to increase the likelihood of negative margins or decrease conversion to mastectomy for DCIS [45].

Management Strategies

The primary goal of DCIS treatment is to keep DCIS from progressing to invasive breast carcinoma. DCIS treatment strategies include surgery (mastectomy or BCS), RT, and adjuvant endocrine therapy in eligible patients to reduce the risk of recurrence [46].

Margin Status

Margin analysis and specimen radiography should be used to document a complete resection. A quantitative description of any tumor close to the margin is helpful for pure DCIS treated with BCS and whole-body radiotherapy (WBRT) [46]. Clinical judgment should be used to weigh the risks of excision against the likelihood of recurrence when there is only minimal or focal DCIS involvement near the margin. The optimal margin width for DCIS treated with excision alone (no WBRT) is unknown; however, a margin of at least 2 mm is recommended [45]. The optimal margin width for DCIS with microinvasion (DCIS-M; invasive focus 1 mm) should refer to the DCIS margin definition (2 mm), and systemic therapy utilization should more closely reflect the treatment pattern for pure DCIS than that for invasive carcinoma [47-49].

Adjuvant therapies

Endocrine therapy with Tamoxifen or an aromatase inhibitor may be considered for ER-positive DCIS treated with mastectomy with sentinel lymph node biopsy or breast-conserving therapy (BCT); the benefit of endocrine therapy for ER-negative DCIS is unknown.

Tamoxifen was compared to a placebo in 1,804 women with DCIS undergoing BCS, followed by radiation therapy in the National Surgical Adjuvant Breast and Bowel Project (NSABP) B-24 study [42,43]. Women in the Tamoxifen arm had fewer breast cancer events after five years of treatment (8.2 vs. 13.4%, p=0.0009) than those in the placebo arm [42,43]. Use of Tamoxifen after BCS for DCIS reduced the recurrence of both ipsilateral DCIS (hazard ratio [HR]=0.75) and contralateral DCIS (relative risk [RR]=0.50), as well as ipsilateral invasive disease (HR=0.79) and contralateral invasive disease (RR=0.57), according to a Cochrane review published in 2012. Even though Tamoxifen uses reduced recurrence, this reviewer found no difference in all-cause mortality (RR=1.11) [42,43].

The NSABP Breast Cancer Prevention Trial found that Tamoxifen reduced the occurrence of invasive breast cancer by 75% in patients with atypical ductal hyperplasia. These findings also demonstrated that Tamoxifen significantly reduced the risk of developing invasive breast disease. Similarly, the NSABP B-24 trial discovered a benefit from Tamoxifen in patients with DCIS after BCS and RT [46]. Patients with DCIS who were treated with BCT were randomly assigned to either a placebo or Tamoxifen in that study. Patients who received Tamoxifen had a 3.4% absolute reduction in ipsilateral in-breast tumor recurrence risk (HR, 0.30; 95% CI, 0.21-0.42; P.001) and a 3.2% absolute reduction in contralateral breast cancers (HR, 0.68; 95% CI, 0.48-0.95; P=.023) after a median follow-up of 13.6 years [43,50-53].

According to the findings of the IBIS-II and NSABP-B-35 studies, anastrozole has a comparable benefit as adjuvant treatment for postmenopausal patients with hormone-receptor-positive DCIS treated with BCS and RT, albeit with a different toxicity profile [54,55].

Several prospective randomized trials of pure DCIS have shown that adding whole breast radiation therapy (WBRT) after BCS reduces the rate of in-breast disease recurrence but not distant metastasis-free survival. A meta-analysis of four large multicenter randomized trials confirmed the individual trials’ findings, demonstrating that adding WBRT after BCS for DCIS results in a statistically and clinically significant reduction in ipsilateral breast events (HR, 0.49; 95% CI; 0.410.58, P.00001) [46]. These trials, however, did not demonstrate that the addition of RT improves overall survival [56-60].

Follow-up

Follow-up should include a history and physical examination every 6-12 months for the first five years, then annually, as well as yearly diagnostic mammography [46]. The first follow-up mammogram should be performed 6-12 months after the completion of RT in patients treated with BCT [46]. Patients undergoing endocrine therapy for risk reduction should be monitored in accordance with NCCN recommendations [46].

Trials are currently underway to see if there is a selected favorable-biology DCIS subgroup that does not require surgical excision. The NCCN Panel continues to recommend surgical excision for all DCIS until definitive evidence of the safety of this nonsurgical approach is demonstrated [46].

## Conclusions

DCIS, an increasing diagnosis among patients with breast mass, clearly contributes to the increase in screening programs along with increased awareness. It remains controversial to comment upon the natural course of the disease, whether to call it an underdiagnosed case of invasive breast carcinoma or benign hyperproliferative breast disease. Imaging modalities remain lower on the specificity side and thus require adjunct histopathological correlation. Histopathology confirms the absence of a breach in the basement membrane, making the diagnosis.

The diagnosis is further refined to determine the grade of disease based on various histopathological features, and risk stratification is done. The prognostication of disease is attempted based on various parameters, and this affects the treatment. Among the various available treatment modalities, along with the available adjuvant therapies, it is then solely dependent on the treating surgeon to choose an appropriate modality that benefits the patient. With the evolving treatment options and the increased research on various aspects of the pathogenesis, the disease remains a potential entity in the field of research.
